# Late-onset first epileptic seizure and cerebral small vessel disease: role of juxtacortical white matter lesions

**DOI:** 10.3389/fneur.2024.1508663

**Published:** 2025-01-23

**Authors:** Adrian Nasca, Leo Sokolovič, Oliver Koprda, Patrick Haage, Thorsten Schmidt, Juraj Kukolja

**Affiliations:** ^1^Faculty of Health, Witten/Herdecke University, Witten, Germany; ^2^Department of Neurology and Clinical Neurophysiology, Helios University Hospital Wuppertal, Wuppertal, Germany; ^3^Department of General and Biological Psychology, University of Wuppertal, Wuppertal, Germany; ^4^Department of Radiology, Helios University Hospital Wuppertal, Wuppertal, Germany; ^5^Department of Diagnostic and Interventional Neuroradiology, Helios University Hospital Wuppertal, Wuppertal, Germany

**Keywords:** juxtacortical, cortical voxel-based morphometry, automated segmentation, white matter lesions, late-onset first epileptic seizure

## Abstract

**Objective:**

The cause of late-onset first epileptic seizures (LOFES) in older age is often not readily evident. In absence of probable causes, it has been suggested that cerebral small vessel disease (CSVD), which is common with increasing age, may be crucial. We aimed to further investigate the impact of white matter lesion (WML) burden and distribution pattern on LOFES.

**Methods:**

We retrospectively compared structural MRI of LOFES patients (*n* = 39) aged 60 years or older to controls with a transient ischemic attack (TIA, *n* = 38) and to patient controls (*n* = 35). WML segmentation was performed on FLAIR images using the SPM based automated lesion prediction algorithm of the LST toolbox and careful manual adjustment. Further, a dichotomization of WML was achieved by use of the BIANCA masking function. A voxel-based morphometry (VBM) analysis was additionally performed on T1 weighted sequences using the automated SPM12 based CAT12 software.

**Results:**

Comparing intrapersonal volume ratios adjusted for the effects of gender and age, we found that the WML distribution was shifted to the juxtacortical compartment in LOFES patients. Among several influencing variables a path analysis could additionally show that this juxtacortical weighting of WML was a significant predictor for LOFES (*β* = 0.509, *p* < 0.001). With regard to total WML volume, LOFES and TIA patients did not differ significantly. Compared to TIA group, LOFES patients gray matter volume was regionally decreased in the right pre- and postcentral gyrus.

**Significance:**

By using algorithm-based automated lesion segmentation software tools and VBM analysis we could highlight that a juxtacortical weighting of WML distribution and regionally decreased gray matter volume distinguished LOFES from TIA and PC groups in our sample.

## 1 Introduction

Late-onset epilepsy (LOE) is usually defined as epilepsy first occurring after the age of 60 (1) and accounts for a third of all incident epilepsy requiring treatment (2). While many cases can be attributed to identifiable causes like cortical stroke or neurodegenerative diseases (3, 4), nearly 40% (5) are deemed idiopathic or are presumed to occur due to cerebral small vessel disease (CSVD) (1, 6), although the exact underlying pathophysiological mechanisms remain unknown. Main risk factors for CSVD apart from age are hypertension and diabetes mellitus (7, 8).

In magnetic resonance imaging (MRI), white matter lesions (WML) are typical manifestations of a CSVD varying in their extent from detached lesions to confluent areas (9). As the human brain contains multiple networks of interconnected neurons, damage to white matter is also assumed to be detrimental to cortical gray matter integrity (10). Prior studies have shown that WML lead to cortical atrophy and cortical hypometabolism in patients with dementia and in cognitively normal older controls (11, 12). The WML volume within a hemisphere also correlates with its regional cortical blood volume (12). Depending on their localization, WML have been shown to be related to various geriatric symptoms. For example, frontally distributed WML have been linked to more frequent progressive cognitive decline or gait apraxia (13). Since white matter volume decreases more rapidly than gray matter volume with increasing age (14), its impact on cerebral function is particularly critical in late life (15).

Regarding WML impact on epilepsy, however, heterogeneous results have been published so far: On the one hand, patients with LOE have been described to have more extensive WML compared to controls (2). On the other hand, it has been reported that in patients with late-onset non-lesional focal epilepsy, seizures were not exclusively related to WML load but strongly correlated with hippocampal atrophy (16).

Using semiquantitative rating scales for WML severity, Stösser et al. (17) found that the quantitative extent of WML does not seem to play a solitary role in LOE due to CSVD and focused on potential differences in spatial distribution patterns of WML: According to their results, juxtacortical lesions were associated with focal seizures with impaired awareness in elderly patients with a high cardiovascular risk (17).

Based on the assumption that epileptic seizures are caused by abnormal excessive or synchronous neuronal activity (18), it is likely that strategical, disruptive distributions of WML or regional cortical atrophy may be crucial risk factors for late-onset first epileptic seizures (LOFES).

Hence, we further investigated the specific influence of WML localization on LOFES using volumetric assessment and regional mapping. With these analyses we aimed to identify a ‘typical distribution pattern’ for LOFES. To this aim, WML were identified and categorized using algorithm-based automated lesion and tissue segmentation software tools. Based on the observation that cortical lesions, e.g., due to stroke rather than subcortical lesions are associated with epilepsy (3, 4) we hypothesized that juxtacortical rather than periventricular lesion distribution may be linked to LOFES.

## 2 Methods

### 2.1 Study design and populations

In a retrospective single-center case–control study, data of 112 patients were included. We selected 39 patients (18 women) with a late-onset first epileptic seizure in its clinical appearances defined by the ILAE in 2017 (18) at the age of 60 years or older (LOFES group). The participants had been treated as inpatients at the Department of Neurology and Clinical Neurophysiology at the Helios University Hospital Wuppertal, Germany, between 2015 and 2020 and had neither evidence of potential epileptogenic cortical lesions nor other plausible explanations for the seizures. Patients with a history of epilepsy or psychogenic seizures, neurodegenerative diseases, amyloid angiopathy, hypoxic brain damage, severe electrolyte disorders or other reasons for acutely symptomatic seizures such as inflammatory diseases of the brain, severe hypoglycemia, substance withdrawal or poisoning were excluded by reviewing the diagnosis lists, anamnesis, laboratory findings and the MRI in the specialist discharge report. Only first-time, unprovoked seizures were considered. Among the patients, 20 (51%) experienced a focal impaired awareness seizure, while 3 (8%) were diagnosed with a focal aware seizure. Additionally, 16 patients (41%) presented with a seizure of generalized onset.

In order to compare WML load between different disease entities, two control groups were chosen. The first group comprised 38 patients (19 women) with a transient ischemic attack (TIA) with a clinically apparent cerebrovascular incident without any cortical lesions in FLAIR and T1-weighted MRI (TIA group). The TIA group included 23 (60%) patients with a cerebrovascular event in the middle cerebral artery (MCA) territory and 15 (40%) in the vertebrobasilar (VB) territory. The second group consisted of 35 patients (21 women) without any clinically apparent epileptic or cerebrovascular incident or cortical lesions (patient control (PC) group). These patients received MRI scans, due to vertigo or headache as part of routine clinical diagnostics. Basic demographic data is shown in Table 1.

**Table 1:** Basic demographic data.

Group	Gender	Median age (range; IQR)	Mean age (SD)
LOFES (*n* = 39)	Female (*n* = 18)	78 (60, 88; 13.5)	75.5 (8.80)
	Male (*n* = 21)	77 (62, 89; 8.0)	75.48 (7.92)
TIA (*n* = 38)	Female (*n* = 19)	79 (62, 91; 13.5)	76.84 (9.12)
	Male (*n* = 19)	77 (60, 91; 9.0)	74.89 (7.90)
PC (*n* = 35)	Female (*n* = 21)	79 (60, 90; 12.0)	76.52 (8.77)
	Male (*n* = 14)	78 (60, 89; 15.5)	75.64 (9.65)

WML were identified and segmented in structural FLAIR images by use of the automated *lesion prediction algorithm* (19) as implemented in the LST toolbox (20) based on the Statistical Parametric Mapping (SPM) software (21) and under careful visual control with manual lesion adjustment. This approach ensured that the WML were captured as comprehensively as possible. Investigators performing lesion-marking were not blinded to group membership. WML were further dichotomized into juxtacortical and (remaining) ‘distacortical’ as well as into periventricular and deep lobar WML using the *distance map tool* of the FMRIB Software Library (FSL) (22). To additionally examine possible alterations in cortical gray matter, structural T1-weighted MRI sequences were processed using the automated SPM based Computational Anatomy Toolbox (CAT12) (23) software to compute mean cortical thickness and to perform a cortical voxel-based morphometry (VBM) analysis.

In addition to MRI, the following patient data were collected: age at time of MRI acquisition (≥ 60 years in all cases), gender, presence of diabetes mellitus, hypertension, hypercholesterolemia/dyslipidemia, atrial fibrillation and coronary artery disease. Obesity and smoking were not listed, as it was not included in diagnostic lists as default. The study was authorized by the local ethics committee.

### 2.2 Data acquisition

Structural MRI scans were acquired in 1.5 T scanners (Avanto FIT/ Aera, Siemens Erlangen, Germany) equipped with a 20-channel phased-array head coil and were extracted in Digital Imaging and Communications in Medicine (DICOM) format.

In accordance with the setting of a retrospective study, MRI protocol selection based on the referral indication and thus varied among the groups: It included a 3D ultrafast gradient echo T1 sequence (MPRAGE with TR/TE = 2.200/2.97 msec; voxel size 1.0 × 1.0 × 1.0 mm; 26 LOFES / 4 TIA / 13 PC), a 2D T1-weighted spin echo sequence (SE with TR/TE = 599.0/15.0 msec; voxel size 0.6 × 0.6 × 5.0 mm; 3 LOFES / 1 TIA / 5 PC), a 2D T1-weighted gradient echo incoherent gradient spoiled sequence (FLASH with TR/TE = 354.0/24.76 msec; voxel size 0.4 × 0.4 × 5.0 mm; 10 LOFES / 33 TIA / 17 PC) and a (3D) T2-weighted fluid attenuated turbo spin echo sequence (FLAIR with TR/TE = 9.000/97 msec; voxel size 0.9 × 0.9 × 4.0 mm; 14 LOFES / 34 TIA / 22 PC or 5.000/404 msec; voxel size 0.8 × 0.8 × 1.0 mm; 25 LOFES / 4 TIA / 13 PC).

### 2.3 Image preprocessing

For image preprocessing, we used the Anatomical Processing Script (fsl_anat, BETA version) provided by FSL with FLAIR images as input files. We mainly kept the default settings of preprocessing which included a reorientation of the images to the standard (MNI) orientation, a bias-field correction, a registration to standard space (linear and non-linear), a brain-extraction, a tissue-type segmentation and last a subcortical structure segmentation. The automated cropping was disabled since the FLAIR images did not cover cervical tissue.

Also, the VBM analysis required preprocessing of the T1-weighted sequences. Because voxel resolution has to be better than 5 mm in any dimension, T1-weighted images with a lower resolution obtained from T1-weighted SE or FLASH sequences had to be upsampled. This was done using a B-spline interpolation in SPM. In the following VBM analysis the default options of the standard preprocessing pipeline of CAT12 [version 1715, (23)] were applied. Described very briefly, first an affine regularization was performed based on the SPM12 tissue probability maps. Within the extended preprocessing options, the strength of corrections affecting the affine preprocessing, for example, the local adaptive segmentation or the internal resampling were adjusted by maintaining the default values. A partial segmentation into gray matter, white matter and cerebrospinal fluid was conducted in a modulated normalized space to compensate for volume changes caused by spatial normalization. As recommended, bias, noise and globally intensity corrected T1-weighted images were written in normalized space as well as partial volume effect label image volumes to ensure a quality control. One patient in the LOFES group had to be excluded due to an insufficient imaging resolution.

### 2.4 Image processing

#### 2.4.1 Adjusted automated WML segmentation with LST-LPA

Before running the automated WML segmentation, the raw DICOM scans were converted into Neuroimaging Informatics Technology Initiative format (NIfTI1) using the Statistical Parametric Mapping Software (SPM12, v. 7,771) (21) in MATLAB R2020a (MathWorks Inc., Natick, MA, USA). Lesion segmentation was performed using the *lesion prediction algorithm* (LST-LPA) (19) as implemented in the LST toolbox (LST, version 3.0.0) (20), which required structural FLAIR images only. The algorithm works with a voxel-wise binary regression model with spatially varying intercepts (20). Because the required bias field correction and affine registration was implemented as part of the preprocessing pipeline, no further preprocessing for WML segmentation was necessary. As output, a probability map was computed and a threshold value had to be set to obtain a binarized segmentation file. Considering that the model was trained on the data of multiple sclerosis patients, which have a potentially different volume and distribution of WML, we adjusted the probability threshold for each patient. The thresholds were varied between 0.1 and 0.9 with a step-size of at least 0.05 and the resulting lesion probability maps were carefully inspected for accuracy. To achieve the most accurate detection possible, hereafter the binarized segmented lesions were precisely corrected in MRIcron (v1.0.20190902,). Finally, the total WML volume (tWML) was calculated using the statistical utility of the FMRIB Software Library [FSL, 6.0.3:b862cdd5, (22)].

#### 2.4.2 Localization related dichotomization of WML

After segmentation and volume estimation of the WML, they were dichotomized in order to enable an analysis of their spatial distribution pattern. Following Stösser et al. (17), we were particularly interested in juxtacortical WML (jWML), which were defined as WML localized at a maximum distance of 3 mm from the cortex (17). WML localized at a greater distance from the cortex were specified as ‘distacortical’ (dWML).

Using the preprocessed data as described above, we created an inclusion map of the cortical gray matter using the *masking function* of the fully automated Brain Intensitiy AbNoramlity Classification Algorithm (BIANCA) (31). Since subcortical gray matter was not removed sufficiently in all cases, it was subtracted using the *modulus remainder function* in FSL and – if necessary – additionally by hand.

Next, a *cortex distance map* from the newly created reference *cortex inclusion map* was computed using the *distance map tool* in FSL (22). A threshold at a distance of 3 mm from the cortex was set to dichotomize the white matter into juxtacortical and distacortical by using *fslmaths* (22). After reorientation into MNI space, the volume (in ml) of the juxtacortical and distacortical WML was computed. To account for interindividual differences in brain size and to obtain a relative WML volume, a ratio between the dichotomized WML and the total intracranial volume (e.g., jWML/TIV ratio) and between the dichotomized WML and the total WML volume (e.g., jWML/tWML ratio) was created.

**Figure 1:**
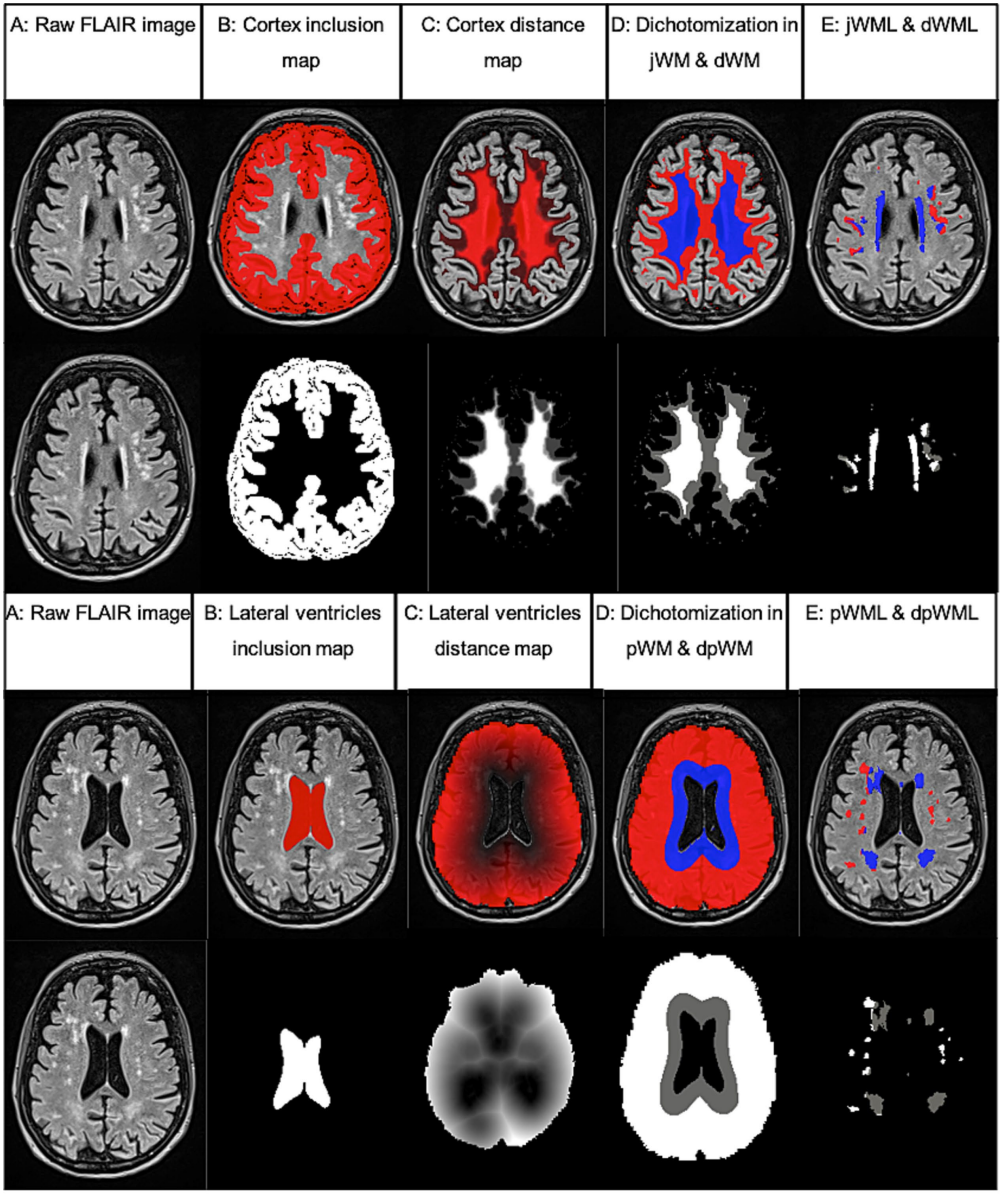
Example of WML segmentation and dichotomization processing steps performed using FSL analysis tools. Based on FLAIR images **(A)** partial inclusion maps **(B)** and referential distance maps **(C)** were calculated and white matter (WM) was dichotomized according to a defined threshold (3 mm from cortex or 10 mm from lateral ventricles, **D**). Last, the volume of the segmented WML within the dichotomized WM was calculated [juxtacortical (jWML, red) and distacortical (dWML, blue) or deep lobar (dpWML, red) and periventricular (pWML, blue) **E**]. Inclusion and distance maps (lower rows) are shown as an overlay on the equivalent FLAIR image in the upper rows.

The same procedure was applied to the categorization into periventricular (pWML) and deep lobar (dpWML) WML as a frequently used dichotomization in literature (10). This time, a reference inclusion map of the lateral ventricles was computed and a threshold was set at a distance of 10 mm from the lateral ventricles wall. All processing steps are shown in Figure 1.

#### 2.4.3 Mean cortical thickness and cortical voxel-based morphometry analysis

The computation of mean cortical thickness and a voxel-based morphometry analysis of cortical gray matter was performed by use of the SPM based Computational Anatomy Toolbox (CAT12) (23).

In brief, all T1-weighted MRI scans were normalized to a template space using an affine registration followed by a non-linear registration, corrected for bias field inhomogeneities and then segmented into gray matter, white matter and cerebrospinal fluid (21). After a quality check by computing and visualizing the correlation between the volumes, image data was smoothed with 8-mm full-width-half-maximum Gaussian smoothing within SPM. The smoothed gray matter images were then analyzed using a 3 (group) x 2 (gender) full factorial analysis. The total intracranial volume (TIV) was used as a nuisance variable. To reduce collinearity, TIV, age at time of MRI and the total amount of WML were mean centered in respect to the respective average of the whole sample and orthogonalized in respect to the two factors in the design matrix. Last, a contrast between LOFES patients and controls was calculated with a voxel-level threshold *p* value of 0.001. The extend threshold was set equal to the expected voxels by cluster (*p*FWE < 0.05). The resulting voxel cluster mask was applied as an overlay on a surface overlay map as implemented in the CAT12 toolbox.

### 2.5 Statistical analyses

Statistical analyses were performed in (R Core Team, 2020). We first report basic demographic data (see Table 1). We then compare the gender and age adjusted risks for diabetes mellitus, hypertension, hypercholesterolemia−/dyslipidemia, atrial fibrillation, coronary artery disease and radiologically reported brain atrophy between the LOFES, TIA and PC groups. To do so, we fitted a generalized linear model with a binomial link function for each variable. The predictors in the model were group, gender and age. The effect of group was then assessed by running an analysis of deviance on the fitted generalized linear model. Next, we compared the three patient groups on the following measures: mean cortical thickness, tWML/TIV ratio, jWML/TIV, pWML/TIV, dWML/TIV, dpWML/TIV, jWML/tWML, pWML/tWML, dpWML/tWML and dWML/tWML ratios as well as the jWML/dWML ratio. The group means were first adjusted for the effects of gender and age using linear regression models. All dependent variables were first transformed to satisfy the normality assumption of the fitted linear regression models. We used power and logarithmic transformations. The assumptions for a linear regression were checked with the help of the *gvlma package* (25). The only exception was the dWML/tWML variable for which a beta regression was fitted. As in all other models, we used group, gender and standardized age as predictors. The beta regression was fit using the *betareg package* (24) Using the fitted models, the estimated marginal means were compared using the *emmeans package* (26), adjusting for the effects of gender and age and applying the Bonferroni correction for multiple comparisons. Plots were created using the *ggplot2* (27) and *patchwork* (28) *packages* for R.

To investigate complex interrelationships between different variables and the risk of a late-onset first epileptic seizure, we used structural equation models, specifically path analyses models and fitted them using the *lavaan R package* (29). We then looked for the model which best balanced parsimony, clinical significance, statistical power and interpretability (see Supplementary materials for model specifications). Statistical model comparisons were conducted using the ‘compareFit’ function from the ‘semTools’ (30) package (for results see Supplementary Table 2). Model fit was assessed using the χ^2^ model fit statistic, the Root Mean Square Error of Approximation (RMSEA), the comparative fit index (CFI) and the Tucker Lewis index (TLI). A non-significant χ^2^, RMSEA <0.08, CFI > 0.90 and TLI > 0.90 were considered to denote a good fit.

To increase statistical power and to focus on the prediction of first-time seizures, we merged the TIA and patient controls into one group and constructed the variable *Epileptic seizure*, with 1 denoting LOFES and 0 denoting no seizure. As *lavaan* requires ordered factors as dependent variables, *Epileptic seizure* was defined as an ordinal variable. The model was fit using the unweighted least squares estimator and the nonlinear minimization subject to box constraints optimization routine. All other model fitting options were left at *lavaan* default settings.

## 3 Results

### 3.1 Baseline characteristics

Extended demographic and medical data can be assessed in the Supplementary Table 1. Besides gender and age, cardiovascular risk factors such as presence of Diabetes mellitus, hypertension, hypercholesterolemia−/dyslipidemia, atrial fibrillation and coronary artery disease is listed there. The prevalence of these medical data in the individual groups are shown in Table 2. Obesity and smoking were not systematically registered and are therefore missing. For LOFES group, also information on pathological EEG findings is provided. The analyses of deviance showed no group differences for diabetes mellitus ( $\chi^{2}=1.08,df=2,p=0.58$ ), hypertension ( $\chi^{2}=5.74,df=2,p=0.06$ ), hypercholesterolemia−/dyslipidemia ( $\chi^{2}=2.90,df=2,p=0.23$ ), atrial fibrillation ( $\chi^{2}=0.40,df=2,p=0.82$ ), coronary artery disease ( $\chi^{2}=1.01,df=2,p=0.60$ ) and brain atrophy ( $\chi^{2}=3.20,df=2,p=0.20$ ).

**Table 2:** Prevalences of cardiovascular risk factors in each group.

Group	DM	HTN	HLD	AF	CAD	Brain atrophy
LOFES (*n* = 39)	0.13	0.85	0.41	0.15	0.23	0.23
TIA (*n* = 38)	0.21	0.95	0.39	0.18	0.16	0.13
PC (*n* = 35)	0.14	0.77	0.23	0.14	0.23	0.09

DM = Diabetes mellitus; HTN = hypertension; HLD = hypercholesterolemia/dyslipidemia; AF = atrial fibrillation; CAD = coronary artery disease.

### 3.2 Group differences in dichotomized WML volumes

We first compared the groups on the tWML/TIV ratio and global cortical thickness. The LOFES patients had a higher tWML/TIV ratio than the PC (M_est_ = 0.0081, SE = 0.0014 vs. M_est_ = 0.0029, SE = 0.0005, *t*_(106)_ = 3.42, *p* = 0.003) but not the TIA group (M_est_ = 0.0057, SE = 0.0010). The PC and TIA groups also differed significantly (*t*_(106)_ = −2.47, *p* = 0.046). Regarding the global cortical thickness, we found that the marginal means of the LOFES (M_est_ = 2.55, SE = 0.02) and TIA (M_est_ = 2.61, SE = 0.02) patients as well as the LOFES and PC (M_est_ = 2.52, SE = 0.03) did not differ. The PC group, however, had a lower estimated global cortical thickness than the TIA group (*t*_(106)_ = −0.09, *p* = 0.032).

**Figure 2:**
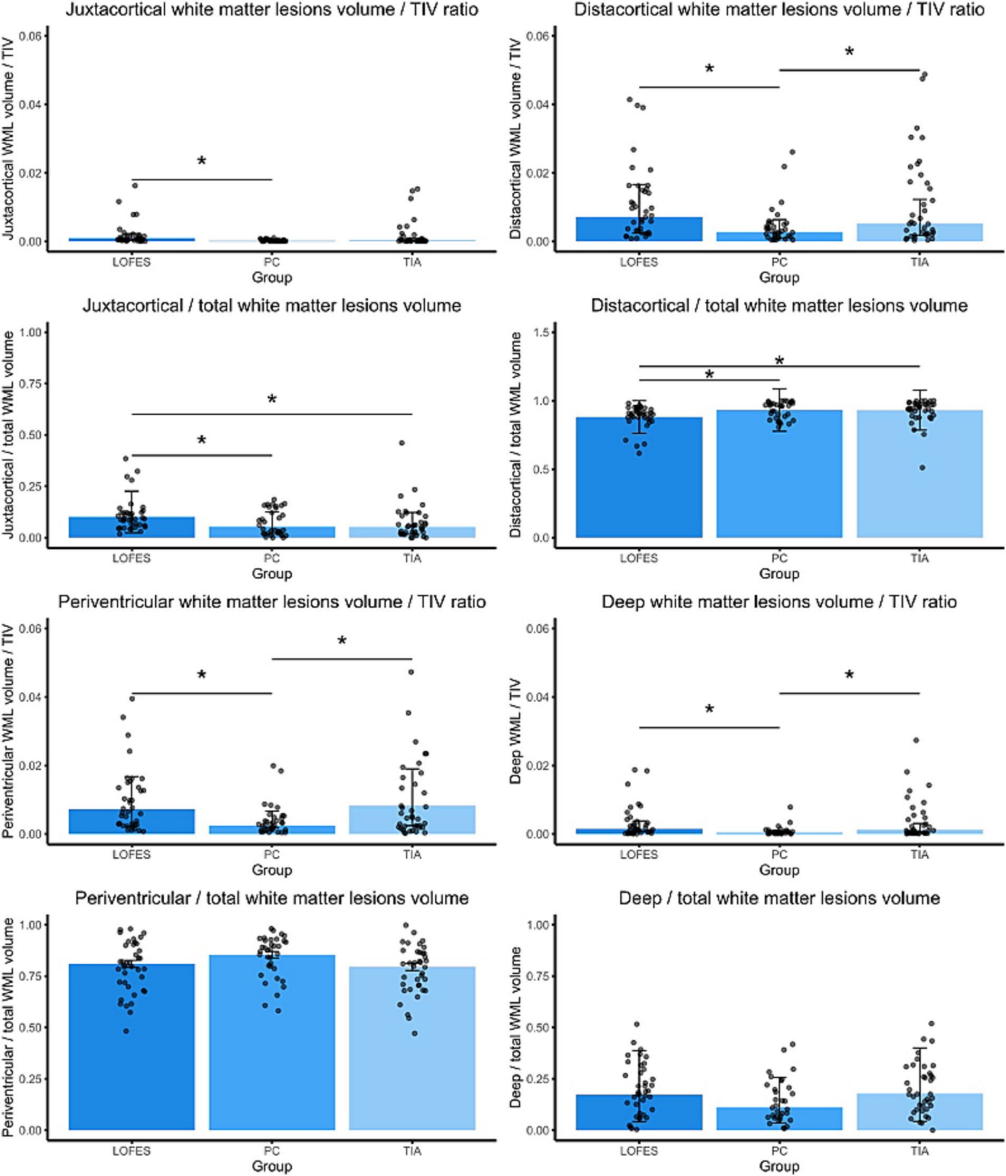
Barplots of estimated marginal means of the dichotomized WML. The stars denote significant differences between estimated group means (*p*_Bonferroni_ < 0.05). A Bonferroni correction for multiple comparisons was applied. Error bars denote one standard error. Dots are the raw data ratios.

Next, we investigated if the LOFES patients differed from the control groups with respect to the ratios of specific WML volumes to TIV and the total WML volume. The comparisons are visualized in Figure 2, while the following text reports the marginal means, t-statistics and *p* values for the comparisons. The comparison of estimated marginal means showed that LOFES patients had a higher jWML/TIV ratio than PC (M_est_ = 0.0009, SE = 0.00022 vs. M_est_ = 0.0001, SE = 0.00004, *t*_(106)_ = 3.54, *p* = 0.002). The LOFES and TIA patients did not differ significantly (M_est_ = 0.0009, SE = 0.00022 vs. M_est_ = 0.0003, SE = 0.00010). Likewise, the PC and TIA groups did not differ significantly. The LOFES group also had a higher jWML/tWML ratio than the PC (M_est_ = 0.010, SE = 0.012 vs. M_est_ = 0.053, SE = 0.009, *t*_(107)_ = 3.18, *p* = 0.006) and the TIA group (M_est_ = 0.052, SE = 0.009, *t*_(107)_ = 3.31, *p* = 0.004). The PC and TIA groups did not differ significantly.

Further, the LOFES group had a higher dWML/TIV ratio than the PC (M_est_ = 0.0071, SE = 0.0012 vs. M_est_ = 0.0027, SE = 0.0005, *t*_(106)_ = 3.34, *p* = 0.003) but not the TIA group (M_est_ = 0.0524, SE = 0.0009). There was also a significant difference between the PC and TIA groups (*t*_(106)_ = −2.48, *p* = 0.043). Likewise, the LOFES patients had a lower dWML/tWML ratio than the PC (M_est_ = 0.88, SE = 0.12 vs. M_est_ = 0.93, SE = 0.15, *z* = −3.34, *p* = 0.002) and the TIA group (M_est_ = 0.93, SE = 0.15, *z* = −3.45, *p* = 0.002). The PC and TIA groups did not differ significantly.

With respect to the pWML/TIV ratio, the LOFES patients had a higher ratio than the PC (M_est_ = 0.007, SE = 0.001 vs. M_est_ = 0.002, SE = 0.001, *t*_(106)_ = 3.67, *p* = 0.001) but not the TIA group (M_est_ = 0.008, SE = 0.001). There was also a significant difference between the PC and TIA groups (*t*_(106)_ = −4.32, *p* < 0.001). We found no differences between the estimated marginal means of groups for the pWML/tWML ratio (M_LOFES_ = 0.81, SE_LOFES_ = 0.02; M_PC_ = 0.85, SE_PC_ = 0.02; M_TIA_ = 0.79, SE_TIA_ = 0.02).

The dpWML/TIV ratio of the LOFES patients was higher than of the PC (M_est_ = 0.0015, SE = 0.0004 vs. M_est_ = 0.0003, SE = 0.0001, *t*_(106)_ = 3.04, *p* = 0.009) but not the TIA group (M_est_ = 0.0012, SE = 0.0003). There was also a significant difference between the PC and TIA groups (*t*_(106)_ = −2.69, *p* = 0.025). We found no differences between the estimated marginal means of groups for the dpWML/tWML ratio (M_LOFES_ = 0.17, SE_LOFES_ = 0.02; M_PC_ = 0.11, SE_PC_ = 0.02; M_TIA_ = 0.18, SE_TIA_ = 0.02).

**Figure 3:**
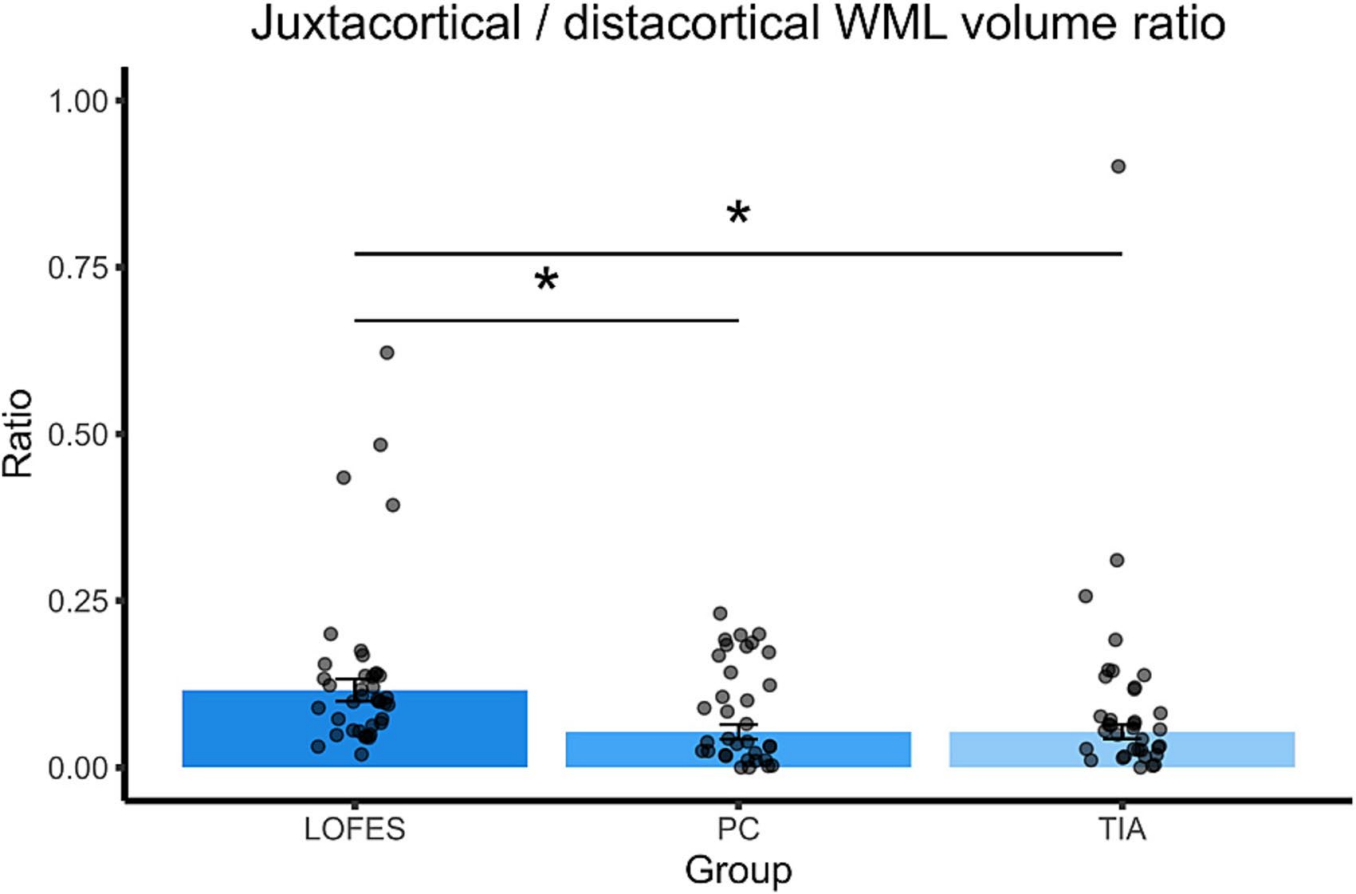
Barplots of estimated marginal means of the juxtacortical/distacortical WML volume ratio. The stars denote significant differences between estimated group means (*p*_Bonferroni_ < 0.05). A Bonferroni correction for multiple comparisons was applied. Error bars denote one standard error. Dots are the raw data ratios.

The LOFES patients also had a higher jWML/dWML ratio than the PC (M_est_ = 0.12, SE = 0.02 vs. M_est_ = 0.05, SE = 0.01, *t*_(107)_ = 3.17, *p* = 0.006) and the TIA group (M_est_ = 0.05, SE = 0.01, *t*_(107)_ = 3.21, *p* = 0.005). The PC and TIA groups did not differ significantly (see also Figure 3). In summary, the analyses showed that LOFES patients had the highest ratio of juxtacortical to total WML volume and the jWML/dWML ratio.

### 3.3 Path analysis

**Figure 4:**
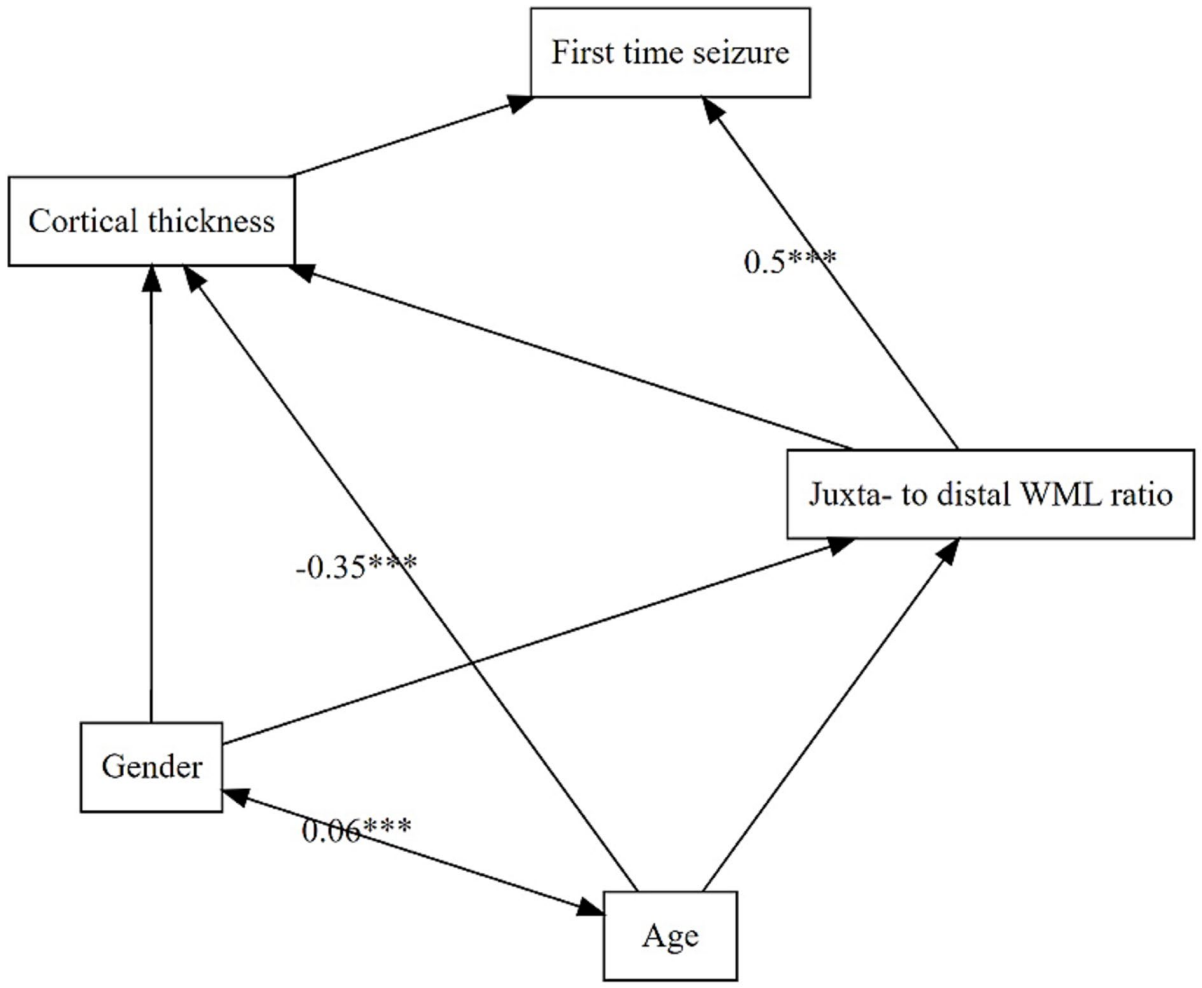
Structural equation model of predictor variables and their impact on each other. Dependent variable is LOFES. Double-headed arrows are mark an intercorrelation whereas single-headed arrows indicate a prediction. Sign. Code ‘***’ 0.001. Root Mean Square Error of Approximation (RMSEA) 0.03 (90% Confidence Interval 0.00, 0.19); Tucker-Lewis Index (TLI) 0.98; Comparative Fit Index (CFI) 0.99 and *X*^2^ (*p*-value) 0.61 (0.62).

The model comparison of our path analysis models indicated that model one best fitted the data (χ^2^ = 0.61, *df* = 2, *p* = 0.62; RMSEA = 0.03, 90% CI [0, 0.19]; CFI = 0.99; TLI = 0.98, see also Supplementary Table 3). It included epilepsy as the dependent variable, cortical thickness and jWML/dWML ratio were endogenous and age and gender the exogenous variables (see Figure 4). The model showed that, in our sample, the only significant predictor of a first time seizure was the juxtacortically shifted ratio jWML/dWML ratio (*β* = 0.509, SE = 0.102, *z* = 4.998, *p* < 0.001). The model also showed that higher age increased global cortical atrophy (*β* = −0.348, SE = 0.097, *z* = −3.577, *p* < 0.001). Model six also contained the pWML/tWML ratio as a predictor of a first time seizure. This model did not prove to be a good model for our data (χ^2^ = 5.18, *df* = 3, *p* = 0.12; RMSEA = 0.16, 90% CI [0.08, 0.25], CFI = 0.34, TLI = 0.50). Further, the path coefficient from the pWML/tWML ratio to first seizure was not significant (*β* = −0.089, SE = 0.124, *z* = −0.719, *p* = 0.472).

### 3.4 Cortical voxel-based morphometry analysis

**Figure 5:**
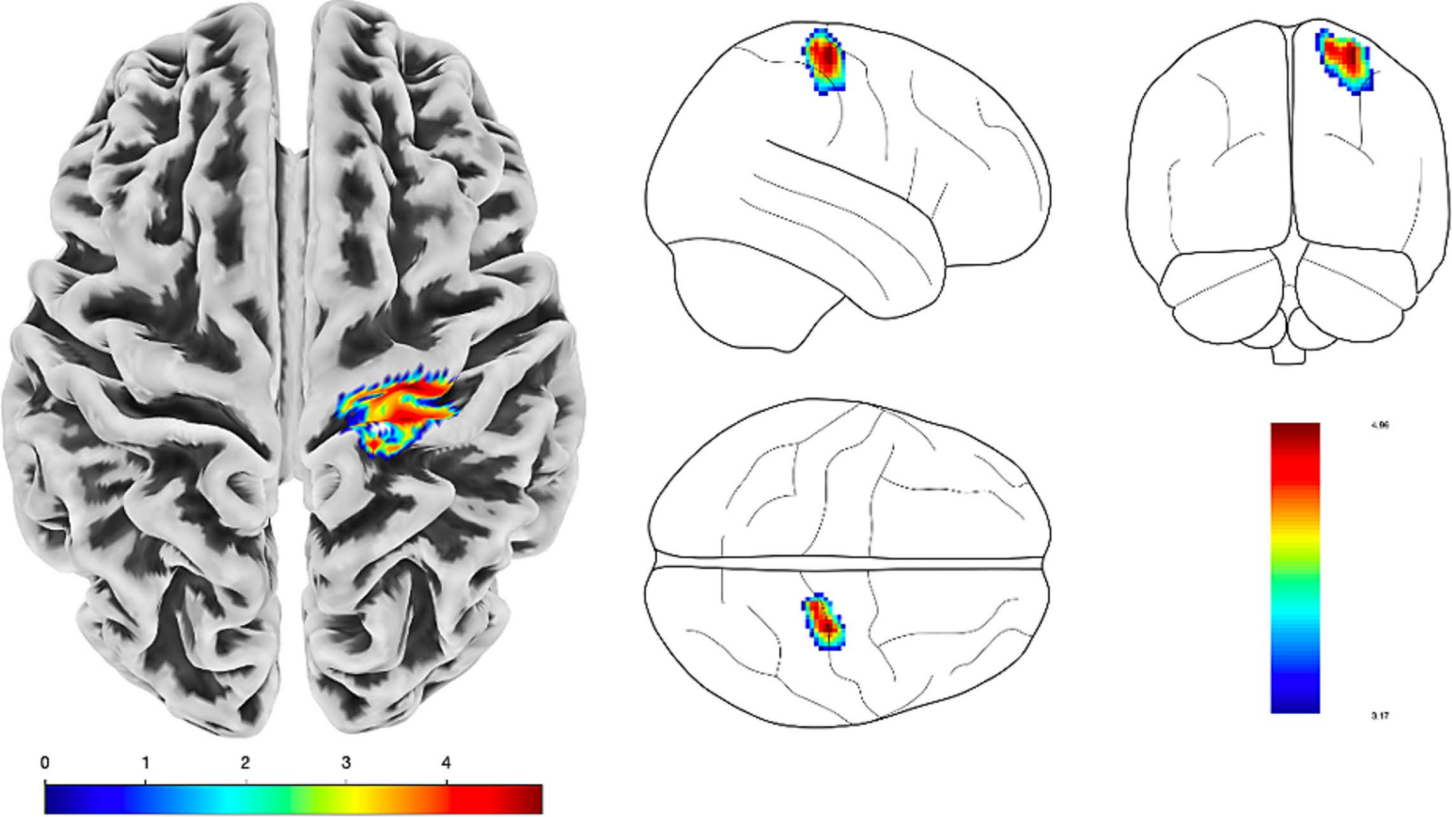
VMB analysis using the CAT12 pipeline. LOFES patients showed a gray matter reduction in the right precentral and post central gyrus compared to TIA patients (*p* < 0.001). The statistical inference (SPM {t}) is presented on a surface overlay map in a top-view and neurological (right side on right side) orientation. Color bars denote *t*-values.

In order to assess between-group differences in regional cortical gray matter volume, we conducted a VBM analysis. LOFES patients showed a significantly decreased gray matter volume in the right precentral and postcentral gyrus when compared to TIA patients (see Figure 5). Anatomical regions were derived from the Neuromorphometrics atlas as implemented in CAT12 by default.

## 4 Discussion

Using the fully-automated *LST – lesion prediction* algorithm (20), the BIANCA *masking function* (31) and extensive manual quality control as a novel and precise approach, we found that the spatial distribution pattern of WML influences late-onset first epileptic seizures with an assumed origin in CSVD.

By dichotomizing cerebral white matter, we found evidence that patients with a late-onset first epileptic seizure showed an juxtacortical predominance of WML compared to controls with and without a clinically manifest cerebrovascular incident (TIA group and patient controls). Moreover, using a path analysis we were able to show that – among several other potential influencing factors – this juxtacortical WML emphasis significantly increases the probability of a first epileptic seizure in older age. This finding is consistent with a previously published study of Stösser et al. (17), who also identified juxtacortical localized small white matter lesions as an independent predictor of focal impaired awareness seizures compared to TIA patients by use of semiquantitative rating scales. As a considerable difference to the mentioned study, we could not detect any significant disparities in LOFES patients compared to TIA patients when WML were subdivided into periventricular and deep lobar WML. Remarkably, the same applied to the total WML load, whose significance in late-onset epilepsy has been controversially debated in literature: While some studies suggest an association between more WML and an increased likelihood of late-onset epilepsy (32), others have reported a lower degree of leukoaraiosis in epilepsy patients compared to controls with a TIA or lacunar stroke (16).

These discrepancies might reflect methodological differences. Instead of using semiquantitative rating scales, we performed an algorithm-based assessment of WML with the aim of a precise, quantitative, interval scaled volumetric determination. Hence, based on the evidence that WML are related to cerebrovascular diseases such as a transient ischemic attack (33, 34), a lack of differences in total WML volume between LOFES and TIA patients does not seem unreasonable. Especially against this background, the pathophysiological and diagnostic significance of a juxtacortical weighting of WML in LOFES patients might become even more important.

Epileptic seizures caused by white matter lesions at first glance seem to be incongruent to our conception of seizures occurring due to pathologically excessive or synchronous neuronal activity (18). However, damage to white matter is assumed to be detrimental to cortical functional efficiency as well (10). Because of a common blood supply with the nearby cortex (Duvernoy type 5, (35)), this might be particularly applicable to the juxtacortical area, as vascular damage in this area may also damage cortical integrity. Analogously, cortical thinning has been related to the presence of juxtacortical lesions in patients with a clinically isolated syndrome or relapsing–remitting MS (36). Stösser et al. (17) hypothesized that juxtacortical small lesions might serve as a surrogate marker for cortical microinfarcts which – due to insufficient image resolution – are not visible in 1.5 T MRI but are common in patients with a cerebral small vessel disease (17, 37). As a consequence, these cortical microinfarcts may decrease the interneural connectivity and disrupt structural networks just as is has been described in cognitively impaired patients (38).

There is a growing body of evidence that epilepsies are network level disorders (36) with either synchronized pathological networks or pathologically synchronized physiological networks (39, 40). Recent studies suggest that the precentral and postcentral gyri may be part of such an epileptogenic network: In temporal lobe epilepsy, for example, volume loss has been observed in bilateral precentral and postcentral gyri, with more severe atrophy associated with frequent seizures (41). Additionally, intraoperative neurophysiologic techniques have been successfully used to guide resection of epileptogenic lesions in the precentral gyrus, highlighting its involvement in epilepsy (42). In accordance with these findings, our VBM analysis showed that the LOFES patients had a decreased gray matter volume in the right precentral and postcentral gyrus compared to TIA patients.

Cerebral small vessel disease is well known to cause brain parenchymal changes, including not only white matter lesions but also cortical microinfarcts (CMIs). Considering the juxtacortical predominance of WML, it seems plausible that such CMIs could be responsible for the defined cortical atrophy. Indeed, an MRI study has already demonstrated that CMIs are linked to perilesional cortical atrophy, exceeding beyond the CMI core and affecting a larger cortical area (43).

However, and somehow highlighting the impact of a juxtacortical white matter lesions predominance on late onset first epileptic seizures with an unknown origin, this did not apply to the group comparison with patient controls. LOFES patients also exhibited no reduced mean cortical thickness compared with both control groups.

Limitations of this study besides the known biases inherent to a retrospective design included variable MR imaging protocols with different voxel resolutions which – due to a partly required upsampling – potentially could have caused a *voxel dimension nuisance covariate* (44). This study warrants further research on algorithm-based volumetric assessments of WML in order to delineate their clinical impact.

## 5 Conclusion

Using the fully-automated *LST – lesion prediction algorithm* (20), the BIANCA *masking function* (31), manual quality control and the cortical VBM analysis tool CAT12 (23) as novel combined approaches, we demonstrated that juxtacortical weighted WML increase significantly the risk of late-onset first epileptic seizures of unknown origin. In contrast, (regional) cortical atrophy could not be detected in comparison to both control groups, which may further emphasize the epileptogenic impact of white matter alterations.

## Data Availability

The raw data supporting the conclusions of this article will be made available by the authors, without undue reservation.
