# Supplementary material for: Late-onset first epileptic seizure and cerebral small vessel disease: role of juxtacortical white matter lesions
Source: Front Neurol. 2025 Jan 23;15:1508663. doi: 10.3389/fneur.2024.1508663 (PMC11799258; doi:10.3389/fneur.2024.1508663)
Supplement: Supplementary file 1 [file Table_1.DOCX]

| **ID** | **group** | **age** | **gender** | | | **SE/PDX** | **EEG/pathological findings** | **DM** | | **HTN** | | **HLD** | | **AF** | | **CAD** | | **brain atrophy** | |
| --- | --- | --- | --- | --- | --- | --- | --- | --- | --- | --- | --- | --- | --- | --- | --- | --- | --- | --- | --- |
| 1 | LOFES | 83 | | male | FIAS | | focal slowing in the theta/delta range in right fronto-temporal region | | 0 | | 1 | | 0 | | 0 | | 0 | | 1 |
| 2 | LOFES | 65 | female | | | FIAS | focal spikes and sharp waves in left temporal region | 0 | | 0 | | 0 | | 1 | | 0 | | 0 | |
| 3 | LOFES | 79 | male | | | BCTS | no pathological findings | 0 | | 1 | | 1 | | 0 | | 1 | | 0 | |
| 4 | LOFES | 85 | female | | | FIAS | focal spikes and sharp waves in left temporal region | 0 | | 1 | | 0 | | 0 | | 0 | | 1 | |
| 5 | LOFES | 77 | male | | | FIAS | no pathological findings | 0 | | 1 | | 1 | | 0 | | 1 | | 0 | |
| 6 | LOFES | 84 | female | | | BCTS | no pathological findings | 0 | | 1 | | 0 | | 0 | | 0 | | 0 | |
| 7 | LOFES | 66 | male | | | BCTS | no pathological findings | 0 | | 0 | | 0 | | 0 | | 0 | | 0 | |
| 8 | LOFES | 82 | male | | | BCTS | no pathological findings | 0 | | 1 | | 0 | | 0 | | 1 | | 0 | |
| 9 | LOFES | 78 | female | | | FIAS | no pathological findings | 1 | | 1 | | 0 | | 0 | | 0 | | 0 | |
| 10 | LOFES | 80 | male | | | BCTS | focal slowing in the theta/delta range with emphasis in right temporoparietal region | 0 | | 0 | | 0 | | 0 | | 0 | | 0 | |
| 11 | LOFES | 86 | female | | | FIAS | generalized epileptiform discharges | 0 | | 1 | | 0 | | 1 | | 0 | | 0 | |
| 12 | LOFES | 87 | male | | | FIAS | no pathological findings | 0 | | 1 | | 0 | | 0 | | 0 | | 1 | |
| 13 | LOFES | 84 | female | | | FIAS | focal spike waves in right temporo-parietal region | 0 | | 0 | | 1 | | 0 | | 0 | | 0 | |
| 14 | LOFES | 78 | male | | | FIAS | no pathological findings | 1 | | 1 | | 1 | | 0 | | 1 | | 0 | |
| 15 | LOFES | 81 | female | | | FAS | focal slowing in the theta/delta range in left temporal region | 0 | | 1 | | 0 | | 0 | | 0 | | 0 | |
| 16 | LOFES | 75 | male | | | BCTS | no pathological findings | 0 | | 1 | | 1 | | 0 | | 1 | | 0 | |
| 17 | LOFES | 79 | female | | | FAS | generalized epileptiform discharges | 0 | | 1 | | 1 | | 0 | | 0 | | 0 | |
| 18 | LOFES | 88 | female | | | FIAS | focal spike waves in right occipital region | 0 | | 1 | | 1 | | 0 | | 0 | | 0 | |
| 19 | LOFES | 94 | female | | | FIAS | focal slowing in the theta/delta range in temporal region on both sides | 0 | | 1 | | 0 | | 0 | | 0 | | 0 | |
| 20 | LOFES | 79 | female | | | FIAS | focal spike waves in left fronto-temporal region | 0 | | 1 | | 0 | | 0 | | 0 | | 0 | |
| 21 | LOFES | 89 | male | | | FIAS | generalized slowing with predominant theta and delta activity | 0 | | 1 | | 1 | | 0 | | 1 | | 0 | |
| 22 | LOFES | 77 | male | | | BCTS | no pathological findings | 0 | | 0 | | 0 | | 0 | | 0 | | 1 | |
| 23 | LOFES | 73 | male | | | BCTS | no pathological findings | 0 | | 1 | | 1 | | 1 | | 0 | | 1 | |
| 24 | LOFES | 87 | female | | | FIAS | focal slowing in the theta range in left parietal region | 0 | | 1 | | 1 | | 1 | | 0 | | 0 | |
| 25 | LOFES | 73 | female | | | BCTS | no pathological findings | 0 | | 1 | | 0 | | 0 | | 0 | | 0 | |
| 26 | LOFES | 80 | female | | | FIAS | no pathological findings | 1 | | 1 | | 0 | | 0 | | 0 | | 0 | |
| 27 | LOFES | 72 | male | | | BCTS | no data available | 0 | | 1 | | 1 | | 0 | | 0 | | 0 | |
| 28 | LOFES | 60 | female | | | BCTS | no pathological findings | 0 | | 1 | | 0 | | 0 | | 0 | | 0 | |
| 29 | LOFES | 86 | male | | | FAS | generalized slowing with predominant theta and delta activity | 0 | | 1 | | 1 | | 1 | | 0 | | 1 | |
| 30 | LOFES | 70 | male | | | FIAS | no pathological findings | 0 | | 1 | | 0 | | 0 | | 0 | | 1 | |
| 31 | LOFES | 79 | male | | | FIAS | no pathological findings | 0 | | 0 | | 1 | | 0 | | 0 | | 1 | |
| 32 | LOFES | 87 | female | | | FIAS | no pathological findings | 0 | | 1 | | 0 | | 0 | | 0 | | 0 | |
| 33 | LOFES | 78 | female | | | BCTS | no pathological findings | 0 | | 1 | | 0 | | 1 | | 0 | | 0 | |
| 34 | LOFES | 89 | male | | | FIAS | no pathological findings | 0 | | 1 | | 1 | | 0 | | 1 | | 0 | |
| 35 | LOFES | 64 | male | | | BCTS | no data available | 1 | | 1 | | 0 | | 0 | | 1 | | 0 | |
| 36 | LOFES | 78 | male | | | BCTS | no pathological findings | 0 | | 1 | | 0 | | 0 | | 0 | | 0 | |
| 37 | LOFES | 75 | female | | | BCTS | generalized epileptiform discharges | 0 | | 1 | | 1 | | 0 | | 0 | | 0 | |
| 38 | LOFES | 72 | male | | | FIAS | no pathological findings | 0 | | 1 | | 0 | | 0 | | 0 | | 0 | |
| 39 | LOFES | 71 | male | | | BCTS | no pathological findings | 1 | | 1 | | 1 | | 0 | | 1 | | 1 | |
| 40 | TIA | 85 | female | | | MCA-TIA |  | 0 | | 1 | | 1 | | 1 | | 1 | | 0 | |
| 41 | TIA | 80 | male | | | MCA-TIA |  | 0 | | 1 | | 1 | | 1 | | 0 | | 1 | |
| 42 | TIA | 66 | female | | | MCA-TIA |  | 0 | | 1 | | 1 | | 0 | | 0 | | 0 | |
| 43 | TIA | 79 | male | | | MCA-TIA |  | 1 | | 1 | | 1 | | 1 | | 1 | | 1 | |
| 44 | TIA | 82 | female | | | MCA-TIA |  | 0 | | 1 | | 0 | | 1 | | 0 | | 0 | |
| 45 | TIA | 77 | male | | | MCA-TIA |  | 0 | | 1 | | 0 | | 0 | | 0 | | 0 | |
| 46 | TIA | 65 | male | | | VB-TIA |  | 0 | | 1 | | 1 | | 0 | | 0 | | 0 | |
| 47 | TIA | 64 | female | | | MCA-TIA |  | 0 | | 1 | | 1 | | 0 | | 0 | | 0 | |
| 48 | TIA | 67 | male | | | MCA-TIA |  | 1 | | 1 | | 1 | | 0 | | 0 | | 0 | |
| 49 | TIA | 80 | male | | | MCA-TIA |  | 0 | | 1 | | 1 | | 1 | | 1 | | 0 | |
| 50 | TIA | 78 | female | | | MCA-TIA |  | 0 | | 0 | | 0 | | 0 | | 0 | | 0 | |
| 51 | TIA | 77 | male | | | MCA-TIA |  | 0 | | 1 | | 1 | | 0 | | 0 | | 0 | |
| 52 | TIA | 85 | female | | | VB-TIA |  | 0 | | 1 | | 0 | | 0 | | 0 | | 0 | |
| 53 | TIA | 91 | female | | | MCA-TIA |  | 0 | | 1 | | 0 | | 0 | | 0 | | 0 | |
| 54 | TIA | 64 | female | | | MCA-TIA |  | 0 | | 1 | | 1 | | 0 | | 0 | | 0 | |
| 55 | TIA | 78 | male | | | MCA-TIA |  | 0 | | 1 | | 0 | | 0 | | 0 | | 0 | |
| 56 | TIA | 82 | female | | | MCA-TIA |  | 1 | | 1 | | 0 | | 1 | | 0 | | 0 | |
| 57 | TIA | 75 | male | | | MCA-TIA |  | 0 | | 1 | | 0 | | 0 | | 0 | | 0 | |
| 58 | TIA | 79 | female | | | MCA-TIA |  | 0 | | 1 | | 0 | | 0 | | 0 | | 0 | |
| 59 | TIA | 80 | female | | | VB-TIA |  | 0 | | 1 | | 0 | | 0 | | 0 | | 0 | |
| 60 | TIA | 75 | female | | | VB-TIA |  | 0 | | 1 | | 0 | | 0 | | 0 | | 0 | |
| 61 | TIA | 63 | male | | | VB-TIA |  | 0 | | 1 | | 1 | | 0 | | 0 | | 0 | |
| 62 | TIA | 60 | male | | | VB-TIA |  | 0 | | 1 | | 0 | | 0 | | 1 | | 0 | |
| 63 | TIA | 75 | male | | | MCA-TIA |  | 0 | | 1 | | 0 | | 0 | | 0 | | 0 | |
| 64 | TIA | 87 | female | | | VB-TIA |  | 0 | | 1 | | 0 | | 0 | | 0 | | 1 | |
| 65 | TIA | 74 | female | | | MCA-TIA |  | 1 | | 1 | | 0 | | 0 | | 0 | | 0 | |
| 66 | TIA | 82 | female | | | MCA-TIA |  | 0 | | 1 | | 0 | | 1 | | 0 | | 0 | |
| 67 | TIA | 75 | male | | | MCA-TIA |  | 0 | | 1 | | 0 | | 0 | | 0 | | 0 | |
| 68 | TIA | 62 | female | | | VB-TIA |  | 1 | | 1 | | 1 | | 0 | | 0 | | 0 | |
| 69 | TIA | 62 | female | | | VB-TIA |  | 0 | | 0 | | 1 | | 0 | | 0 | | 0 | |
| 70 | TIA | 91 | male | | | VB-TIA |  | 0 | | 1 | | 0 | | 0 | | 0 | | 1 | |
| 71 | TIA | 81 | male | | | VB-TIA |  | 0 | | 1 | | 0 | | 0 | | 0 | | 0 | |
| 72 | TIA | 63 | male | | | VB-TIA |  | 1 | | 1 | | 0 | | 0 | | 0 | | 0 | |
| 73 | TIA | 81 | male | | | MCA-TIA |  | 1 | | 1 | | 0 | | 0 | | 0 | | 1 | |
| 74 | TIA | 77 | female | | | MCA-TIA |  | 0 | | 1 | | 1 | | 0 | | 0 | | 0 | |
| 75 | TIA | 81 | male | | | MCA-TIA |  | 0 | | 1 | | 0 | | 0 | | 1 | | 0 | |
| 76 | TIA | 85 | female | | | VB-TIA |  | 1 | | 1 | | 0 | | 0 | | 1 | | 0 | |
| 77 | TIA | 75 | male | | | VB-TIA |  | 0 | | 1 | | 1 | | 0 | | 0 | | 0 | |
| 78 | PC | 81 | male | | | syncope |  | 0 | | 1 | | 0 | | 0 | | 0 | | 0 | |
| 79 | PC | 83 | female | | | headache |  | 1 | | 1 | | 0 | | 0 | | 0 | | 0 | |
| 80 | PC | 85 | male | | | vertigo |  | 0 | | 1 | | 0 | | 0 | | 0 | | 0 | |
| 81 | PC | 65 | female | | | vertigo |  | 0 | | 1 | | 0 | | 0 | | 0 | | 0 | |
| 82 | PC | 79 | female | | | vertigo |  | 1 | | 1 | | 0 | | 0 | | 0 | | 0 | |
| 83 | PC | 68 | female | | | vertigo |  | 0 | | 0 | | 0 | | 0 | | 0 | | 0 | |
| 84 | PC | 64 | male | | | headache |  | 0 | | 0 | | 0 | | 0 | | 0 | | 0 | |
| 85 | PC | 77 | female | | | vertigo |  | 0 | | 1 | | 0 | | 1 | | 0 | | 0 | |
| 86 | PC | 78 | male | | | vertigo |  | 0 | | 0 | | 1 | | 0 | | 0 | | 0 | |
| 87 | PC | 85 | female | | | vertigo |  | 0 | | 1 | | 1 | | 1 | | 1 | | 0 | |
| 88 | PC | 64 | female | | | vertigo |  | 0 | | 0 | | 0 | | 0 | | 0 | | 0 | |
| 89 | PC | 77 | male | | | vertigo |  | 0 | | 1 | | 0 | | 0 | | 0 | | 0 | |
| 90 | PC | 86 | female | | | vertigo |  | 0 | | 1 | | 0 | | 0 | | 1 | | 0 | |
| 91 | PC | 79 | female | | | vertigo |  | 0 | | 1 | | 1 | | 1 | | 0 | | 0 | |
| 92 | PC | 85 | female | | | vertigo |  | 0 | | 1 | | 0 | | 0 | | 1 | | 0 | |
| 93 | PC | 79 | female | | | headache |  | 1 | | 1 | | 0 | | 0 | | 1 | | 0 | |
| 94 | PC | 71 | female | | | headache |  | 0 | | 0 | | 0 | | 0 | | 0 | | 0 | |
| 95 | PC | 81 | female | | | headache |  | 1 | | 1 | | 1 | | 0 | | 1 | | 0 | |
| 96 | PC | 61 | male | | | headache |  | 0 | | 1 | | 0 | | 0 | | 0 | | 0 | |
| 97 | PC | 60 | male | | | vertigo |  | 0 | | 1 | | 0 | | 0 | | 0 | | 0 | |
| 98 | PC | 83 | female | | | headache |  | 0 | | 1 | | 1 | | 0 | | 0 | | 1 | |
| 99 | PC | 71 | female | | | headache |  | 0 | | 1 | | 0 | | 1 | | 0 | | 0 | |
| 100 | PC | 84 | female | | | vertigo |  | 0 | | 1 | | 1 | | 0 | | 0 | | 0 | |
| 101 | PC | 61 | female | | | vertigo |  | 0 | | 0 | | 0 | | 0 | | 0 | | 0 | |
| 102 | PC | 60 | female | | | vertigo |  | 0 | | 0 | | 0 | | 0 | | 0 | | 0 | |
| 103 | PC | 79 | female | | | headache |  | 0 | | 1 | | 0 | | 0 | | 0 | | 0 | |
| 104 | PC | 89 | male | | | vertigo |  | 0 | | 1 | | 0 | | 0 | | 0 | | 1 | |
| 105 | PC | 78 | male | | | vertigo |  | 0 | | 1 | | 0 | | 0 | | 1 | | 0 | |
| 106 | PC | 87 | male | | | vertigo |  | 0 | | 1 | | 0 | | 0 | | 0 | | 0 | |
| 107 | PC | 65 | male | | | vertigo |  | 0 | | 1 | | 1 | | 0 | | 1 | | 0 | |
| 108 | PC | 83 | male | | | vertigo |  | 0 | | 1 | | 0 | | 0 | | 0 | | 1 | |
| 109 | PC | 77 | female | | | vertigo |  | 0 | | 1 | | 0 | | 0 | | 0 | | 0 | |
| 110 | PC | 78 | male | | | vertigo |  | 0 | | 1 | | 1 | | 1 | | 1 | | 0 | |
| 111 | PC | 90 | female | | | vertigo |  | 1 | | 1 | | 0 | | 0 | | 0 | | 0 | |
| 112 | PC | 73 | male | | | vertigo |  | 0 | | 0 | | 0 | | 0 | | 0 | | 0 | |

Supplementary Tab 1. Extended demographic and medical data of the study population: Please note the following abbreviations: ID = Patient ID; SE/PDX = Seizure type/Primary diagnosis; DM = Diabetes mellitus; HTN = Hypertension; HLD = Hyperlipidemia/Hypercholesterolemia; AF = Atrial fibrillation; CAD = Coronary artery disease; LOFES = Late-onset first epileptic seizure; TIA = Transient ischemic attack; PC = Patient controls; FIAS = Focal impaired awareness seizure; FAS = Focal aware seizure; BTCS = Bilateral tonic-clonic seizure; MCA-TIA = Transient ischemic attack in the territory of the middle cerebral artery; VB-TIA = Transient ischemic attack in the vertebrobasilar territory. 0 = Criterion not met; 1 = Criterion met. Brain atrophy refers to a finding described by a radiologist in the written MRI report as part of routine diagnostics.

## Path analysis models

| Supplementary table 2. Path analysis models | |
| --- | --- |
| Model number | Model specification |
| 0 | # Cortical Thickness  Cortical_Thickness ~ J/D_ratio + Age  # Juxtacortical ratio  J/D_ratio ~ Age  # FES  FES ~ Cortical_Thickness + J/D_ratio |
| 1 | # Cortical thickness  Cortical_Thickness ~ Age + Gender + J/D_ratio  # Juxtacortical ratio  J/D_ratio ~ Age + Gender  # FES  FES ~ Cortical_Thickness + J/D_ratio |
| 2 | # Cortical thickness  Cortical_Thickness ~ Age + Gender + J/D_ratio + WML/TIV  # Juxtacortical ratio  J/D_ratio ~ Age + Gender + WML/TIV  # FES  FES ~ Cortical_Thickness + J/D_ratio + WML/TIV |
| 3 | # Cortical thickness  Cortical_Thickness ~ Age + J/D_ratio + WML/TIV  # Juxtacortical ratio  J/D_ratio ~ Age + WML/TIV  # FES  FES ~ Cortical_Thickness + J/D_ratio + WML/TIV |
| 4 | # Cortical thickness  Cortical_Thickness ~ J/D_ratio + Age  # Juxtacortical ratio  J/D_ratio ~ Age + WML/TIV  # FES  FES ~ Cortical_Thickness + J/D_ratio + WML/TIV |
| 5 | # Cortical thickness  Cortical_Thickness ~ J/D_ratio + Age  # Juxtacortical ratio  J/D_ratio ~ WML/TIV  # FES  FES ~ J/D_ratio + Cortical_Thickness |
| 6* | # Cortical thickness  Cortical_Thickness ~ Age + Gender + J/D_ratio  # Juxtacortical ratio  J/D_ratio ~ Age + Gender  # FES  FES ~ Cortical_Thickness + J/D_ratio + pWML/tWML |
| * this model includes the pWML/tWML ratio, whose inclusion was suggested by a reviewer. | |
| Notes: FES = first ever seizure; J/D_ratio: juxtacortical / distacortical white matter lesion volume ratio; WML/TIV: white matter lesion / total intracranial volume ratio | |

| Supplementary table 3. SEM Model comparison | | | | | | | |
| --- | --- | --- | --- | --- | --- | --- | --- |
| Model # | $\chi^{2}$ | *df* | *p* | RMSEA | CFI | TLI | SRMR |
| **1** | **0.61** | **2** | **0.62** | **0.00** | **1.00** | **1.06** | **0.02** |
| 2 | 0.68 | 2 | 0.61 | 0.00 | 1.00 | 1.08 | 0.02 |
| 5 | 1.57 | 3 | 0.69 | 0.00 | 1.00 | 1.06 | 0.02 |
| 3 | 1.64 | 1 | 0.20 | 0.08 | 0.97 | 0.93 | 0.02 |
| 0 | 1.67 | 1 | 0.20 | 0.08 | 0.97 | 0.94 | 0.02 |
| 4 | 1.96 | 2 | 0.32 | 0.03 | 0.99 | 0.99 | 0.02 |
| 6 | 5.18 | 3 | .12 | 0.10 | 0.84 | 0.88 | 0.02 |
| Note: bold marked model is the selected model. The scaled versions of the root mean squared error of approximation (RMSEA), comparative fit index (CFI), Tucker Lewis index (TLI) and standardized root mean residual (SRMR) are reported. | | | | | | | |


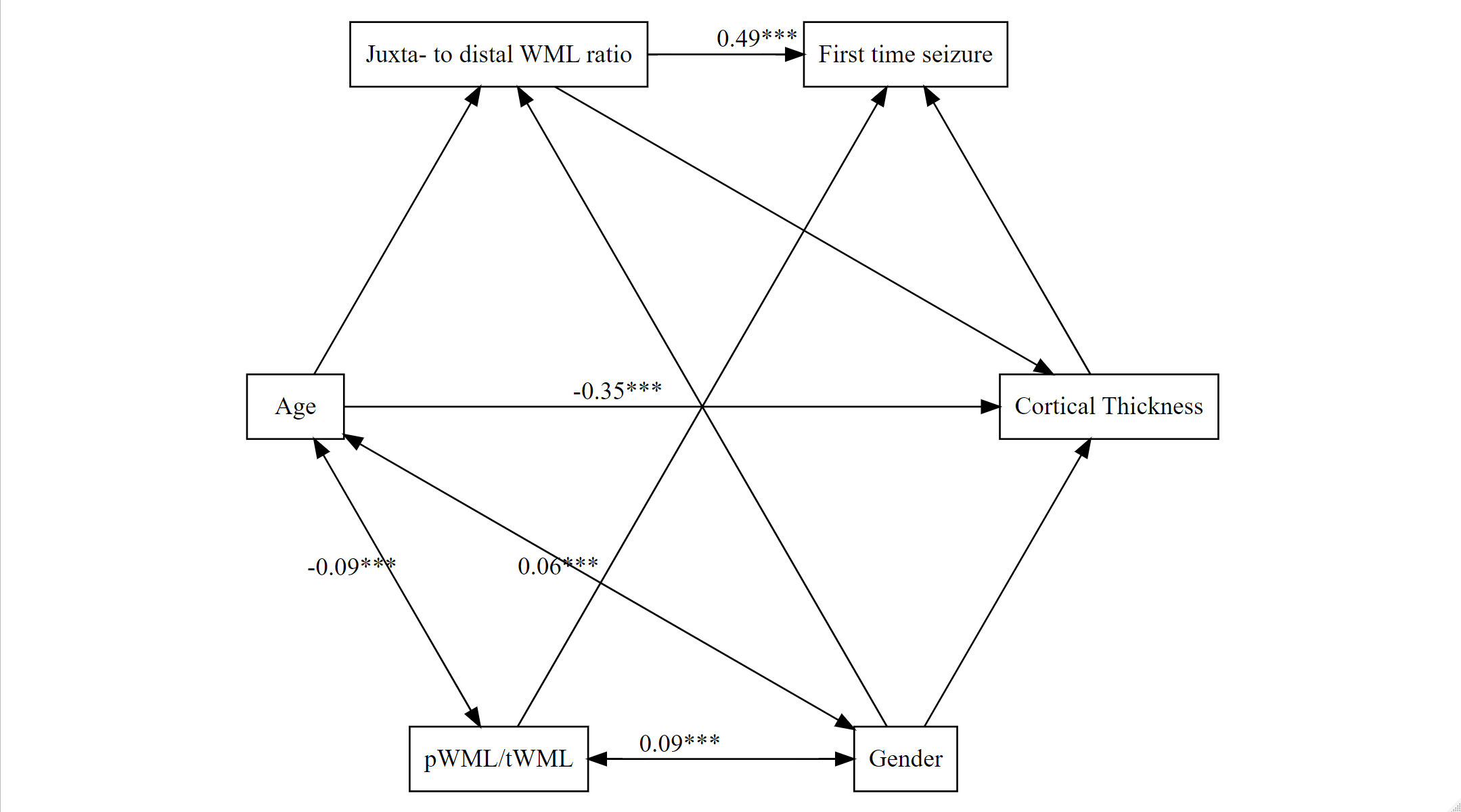


Supplementary figure 1. The path model 6. The addition of the pWML/tWML ratio worsens model fit (see supplementary table 3). The JWML/dWML is still the only significant predictor of a first time seizure.
